# Pilot Study on the Assessment of Therapeutic Education in Children and Adolescents with Type 1 Diabetes Mellitus

**DOI:** 10.3390/healthcare13020109

**Published:** 2025-01-08

**Authors:** Dominik Olejniczak, Vivienne Tworzydlak, Aleksandra Kurowska, Karolina Blaszka, Katarzyna Swakowska, Anna Staniszewska

**Affiliations:** 1 Department of Public Health, Medical University of Warsaw, 02-091 Warsaw, Poland; dominik.olejniczak@wum.edu.pl; 2 Clinic of Pediatric Diabetology and Pediatrics, Polikarp Brudzinski Independent Public Children’s Clinical Hospital in Warsaw, 02-091 Warsaw, Poland; viviennee.tworzydlak@gmail.com; 3 Faculty of Health Sciences, Poznan University of Medical Sciences, Fredry 10, 61-701 Poznan, Poland; 4 Medical Services Records Department, Greater Poland Cancer Centre, Garbary 15, 61-866 Poznan, Poland; karolina.blaszka@wco.pl; 5 Gute-Zeit24 Pflegedienst, 63785 Obernburg am Main, Germany; swakowskakasia@gmail.com; 6 Department of Experimental and Clinical Pharmacology, Medical University of Warsaw, 02-091 Warsaw, Poland; anna.staniszewska@wum.edu.pl

**Keywords:** type 1 diabetes mellitus, therapeutic education, children, diabetes management, disease management

## Abstract

**Introduction:** Type 1 diabetes mellitus (T1DM) is a chronic metabolic disorder primarily managed through insulin therapy, which is crucial for achieving optimal glycemic control in children and adolescents. Therapeutic education is essential, equipping patients and their families with the knowledge and skills necessary for effective self-management. This familial support plays a critical role in the success of therapy and in fostering an environment conducive to the child’s self-management of the condition. A core objective of therapeutic education is the prevention of complications associated with T1DM. Children and adolescents are made aware of the importance of consistent disease management by receiving information about the potential consequences of prolonged metabolic dysregulation. **Methods:** The study employed a diagnostic survey method with a proprietary questionnaire consisting of a demographic section and 28 closed-ended questions. The survey was conducted between February and May 2024 at the Pediatric Diabetes and Pediatrics Clinical Department of the Polikarp Brudziński Independent Public Children’s Clinical Hospital in Warsaw. A total of 100 valid responses were included. Data were analyzed using frequency analysis for categorical variables, descriptive statistics for quantitative data, and Pearson’s Chi-squared test for relationship analysis. **Results:** Most parents (76%) initiated knowledge acquisition in diabetology departments. Group and individual training sessions were attended by 58% of respondents. Training frequency was predominantly daily (88%), and 92% of parents stressed the importance of consistent education. The vast majority (96%) of participants rated the training as well prepared, with 100% affirming that the education was adapted to their needs. **Conclusions:** Therapeutic education for children with T1DM is highly effective, particularly when integrating individual and group training sessions. Regular daily exercise is associated with higher levels of knowledge and skills in diabetes management. This emphasizes the importance of structured and frequent educational programs to optimize disease control, enhance familial support, and prevent complications, ultimately improving patient outcomes.

## 1. Introduction

Type 1 diabetes mellitus (T1DM) is among the most prevalent chronic metabolic disorders, frequently manifesting in children across various age groups. Management primarily involves insulin therapy, necessitated by the absence of endogenous insulin production [1]. The global incidence of T1DM varies geographically, as reflected in epidemiological data. According to the International Diabetes Federation (IDF), 8.75 million individuals (95% uncertainty interval: 8.4–9.1 million) lived with T1DM worldwide in 2022. Notably, 1.9 million (approximately 20%) of these individuals resided in low- and middle-income countries. Of the total T1DM population in 2022, 1.52 million (17%) were under the age of 20, 5.56 million (64%) were between 20 and 59 years old, and 1.67 million (19.9%) were aged 60 years or older. The estimated figure of 1.52 million for individuals under 20 years of age in 2022 represents an increase from the 2021 estimate of 1.21 million. This rise is attributed to the adoption of a novel methodology that accounts for growth in disease incidence over time in countries lacking recent incidence data, as well as modest population growth [2].

Therapeutic education in the management of type 1 diabetes in children and adolescents is as fundamental as pharmacotherapy for achieving optimal disease control. Defining therapeutic education precisely is challenging due to its multifaceted nature [3]. According to the World Health Organization (WHO), therapeutic education is an ongoing, integral part of the treatment process, with the patient at its center. The modern approach to therapeutic education aims to enhance the self-care competencies of patients, their parents, and families through collaboration with interdisciplinary healthcare teams. This process is tailored to individual goals, quality of life, and minimizing the risks associated with type 1 diabetes [4].

Children and adolescents are educated on the importance of the following:

● Regular glucose monitoring;
● Proper insulin administration;
● Dietary adjustments and physical activity management [5].

Therapeutic education emphasizes the development of practical self-care skills, fostering an informed approach to treatment and health-related decision-making. It seeks to adapt diabetes management to the daily lives of children and adolescents, incorporating treatment into various contexts such as school, social interactions, and the family environment. A key component of this process is addressing the psychosocial challenges associated with the disease. Through education, children and adolescents are equipped to manage the emotional aspects that may emerge in their relationships with peers, within the school setting, or in family life. Support for families is also an essential element of the educational framework [5,6].

Effective self-management is the ultimate goal of well-structured and carefully delivered therapeutic education, tailored to the specific needs and perceptual capacities of the target population. This approach is particularly critical in diabetes mellitus, where self-management encompasses not only the therapeutic regimen but also the comprehensive management of the disease, including dietary adherence and appropriate physical activity. This underscores the intrinsic link between self-management and therapeutic education. Studies demonstrate that therapeutic education significantly improves glycemic control, reducing the risk of both acute and long-term complications associated with diabetes [7,8,9].

The WHO emphasizes that effective diabetes education programs can significantly improve glycemic control and the quality of life for individuals with type 1 diabetes. These programs, such as the structured education frameworks for diabetes management, focus on improving patient skills in managing their condition, which is crucial for long-term health outcomes. One key finding that could be included is the significant improvement in HbA1c levels seen in participants following structured diabetes education programs, like the Diabetes Education and Self-Management for Ongoing and Newly Diagnosed (DESMOND) or the Diabetes and Well-being (DAFNE) programs. Studies have shown that such programs not only help control blood sugar levels but also enhance life satisfaction and reduce complications associated with diabetes, which is in line with WHO recommendations for comprehensive diabetes care [10].

Additionally, the WHO advocates for a patient-centered approach to diabetes care that incorporates education and self-management as fundamental components of health system strategies. This approach is particularly relevant when discussing the inclusion of patient organization experiences in evaluating medical technologies, as they can provide valuable insights into the practical outcomes of educational interventions by linking local findings with international best practices in diabetes management and patient care [11].

Therapeutic education equips families with the knowledge and skills necessary to effectively support their child in the daily management of type 1 diabetes. This familial support plays a critical role in the success of therapy and in fostering an environment conducive to the child’s self-management of the condition. Furthermore, a core objective of therapeutic education is the prevention of complications associated with type 1 diabetes. Children and adolescents are informed of the importance of consistent disease management by receiving information about the potential consequences of prolonged metabolic dysregulation [6,12].

This study aimed to evaluate the perceived effectiveness of the educational and therapeutic process, including the relevance of its content, as reported by parents and caregivers of children and adolescents with type 1 diabetes. Additionally, the study sought to elucidate the role of healthcare professionals in delivering effective therapeutic education.

Main Hypothesis: The therapeutic education process in children and adolescents with type 1 diabetes is effective.

## 2. Material and Methods

This research employed a diagnostic survey method, utilizing a questionnaire technique. The research instrument was a proprietary questionnaire, comprising a demographic section and 28 closed-ended questions. The questionnaire was developed based on a theoretical framework of factors influencing the effectiveness of therapeutic education, grounded in the relevant literature reviewed in this publication and the work of Passmore et al. [13].

The survey was conducted at the Pediatric Diabetes and Pediatrics Clinical Department of the Polikarp Brudziński Independent Public Children’s Clinical Hospital in Warsaw between February and May 2024. Before initiating the research, written consent was obtained from the hospital’s Directorate, and ethical approval was granted by the Bioethics Committee at the Medical University of Warsaw (Approval No. AKBE/38/2024) [13].

The study site utilizes an algorithm for therapeutic education designed by experienced educators and grounded in practical insights. The educational process follows distinct stages, including needs assessment, goal setting, education planning, training delivery, and the evaluation of training quality (the primary focus of this study). Consequently, the training duration is adapted to the individual needs of each patient. The hospital environment supports a two-way communication model, enhancing effectiveness by allowing patients to ask questions, clarify uncertainties, and receive repeated instruction, especially when education is delivered through demonstration. Each session lasts approximately 1.5 h.

Participants were fully informed about the purpose of the study and their voluntary involvement. They were also provided with detailed instructions on how to complete the questionnaire and were assured of the anonymity and confidentiality of the data collected. The research was conducted anonymously and voluntarily. A total of 110 questionnaires were distributed, of which 3 were excluded from the sample due to incorrect completion, and 7 were not returned. Ultimately, 100 questionnaires were included in the final analysis.

The data were analyzed using frequency analysis (n, %) for categorical variables. For quantitative data, descriptive statistics such as mean, median, and standard deviation were calculated. Relationships between qualitative variables were examined using Pearson’s Chi-squared (χ^2^) test. A significance level of α = 0.05 was adopted for all statistical analyses.

A key strength of this study is its innovative focus—particularly within the context of Polish healthcare—on therapeutic education, with findings applicable and relevant to international audiences. Additionally, the study addresses the increasingly prevalent issue of pediatric diabetes, highlighting the potential impact of well-planned educational tools on improving treatment outcomes.

A notable limitation of the study is the frequent reluctance of patients, particularly parents, to engage in the educational process. The initial shock of diagnosis can lead to denial, creating barriers to access within this group. Consequently, this pilot study serves as a preliminary investigation, providing groundwork for a more comprehensive analysis currently underway.

## 3. Results

Before their child’s diagnosis, only 22% of parents reported knowing type 1 diabetes. Notably, the majority of parents (76%) began the process of expanding their knowledge in diabetology departments, while 12% indicated that they received such education in a clinic, and 14% cited “other places”. The initiation of this knowledge enhancement process is a highly positive outcome.

Regarding participation in educational programs, 58% of surveyed parents reported attending both individual and group training sessions. Group training alone was attended by 40% of respondents, while only 2% participated exclusively in individual training. 

When asked about the frequency of training, 88% of parents reported receiving training daily, while 12% indicated that training occurred every other day. The intensity of parental training is critically important in the context of managing a child’s illness, as it directly impacts the ability to care for the child and contributes to effective therapy post-hospitalization. Notably, 92% of parents emphasized the importance of regular participation in the training process for type 1 diabetes, underscoring the value that they place on consistent education to support their child’s ongoing care.

Parents also reported receiving an information sheet outlining the training schedule before the commencement of the educational sessions (88.00%), which likely contributed to the regularity of their participation in the education program.

Parents involved in the type 1 diabetes education process were able to identify the professions of the educators (within the teams) conducting the training. The majority (74.00%) indicated that the teams consisted of nurses, dietitians, and physicians. Training sessions led exclusively by nursing teams were reported by 16.00% of respondents, while 10.00% indicated that the training was conducted solely by dietitians.

Training provided by nursing teams was most frequently rated as being of very high quality, with 72.00% of parents assigning an “excellent” rating, while 28.00% evaluated the training as “good”.

Training on type 1 diabetes conducted by a team of dietitians received predominantly positive evaluations, with 64.00% of respondents rating it as “excellent” and 28.00% as “good”. Conversely, 8.00% of respondents assigned a “poor” rating to the training.

In the case of training delivered by physicians, 56.00% rated it as “excellent”, while 30.00% rated it as “good”, and 14.00% assigned a “poor” rating.

Another question addressed the preparation level of the training. The vast majority of participants (96.00%) reported that the training was well prepared, accessible, and useful, with only 2.00% expressing a negative opinion. Notably, all respondents (100%) felt that the trainer effectively adapted the terminology and delivery to meet the needs and perceptual capabilities of the audience. Additionally, 86.00% indicated that the didactic content was tailored to the needs of children.

A significant finding was that 92.00% of respondents believed that the theoretical knowledge presented during the training was effectively translated into practical applications, such as proper injection techniques and glucometer operation.

The training sessions were characterized by a bidirectional communication model, as confirmed by all participants (100%), who reported having the opportunity to ask questions during the sessions. Furthermore, all respondents stated that the answers provided by the trainers were satisfactory (100.00%).

**Figure 1:**
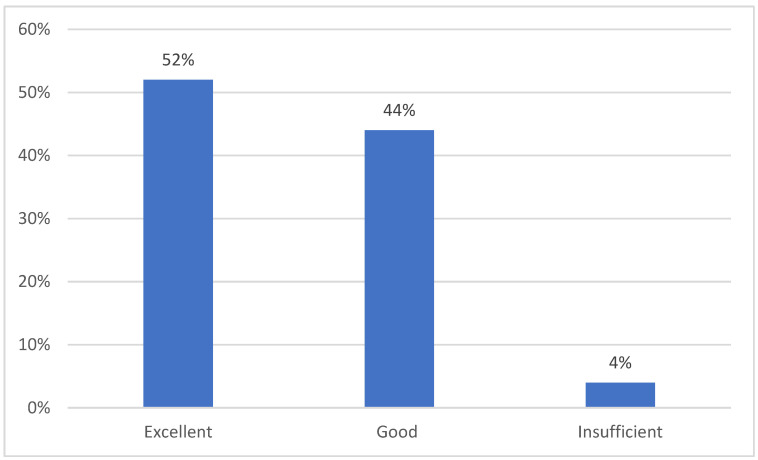
Assessment of the level of knowledge and skills needed for diabetes care after the educational process in the study group (N = 100). Source: Authors’ own elaboration based on the survey.

The proficiency in knowledge and skills necessary for diabetes care was also evaluated among parents following the educational process. An excellent level of knowledge was demonstrated by 52.00% of respondents, while 44.00% exhibited a good level of knowledge. The remaining 4.00% displayed an inadequate level of knowledge (Figure 1).

## 4. Evaluation of Dependencies

The relationship between the forms, place, or frequency of training and skills and knowledge of type I diabetes was assessed. It was observed that among those showing excellent levels of skills, training was most often held in diabetology departments (76.92%; 40 people) and less often in clinics (19.23%; 10 people) or other places (3.85%; 2 people).

Parents showing a good level of skills usually also began their training in diabetology departments (72.73%; 32 people), and less often in other places (22.73%; 10 people). All those showing an inadequate level of knowledge started their education in diabetology departments.

The relationship shown is statistically significant (χ^2^ = 12.73; df = 4; *p* = 0.012) (Table 1).

The relationship between the education model and diabetes skills and knowledge was evaluated. It was shown that in the group of people showing excellent skills, the education model was structured training (92.31%; 48 people). In the group of parents showing good knowledge, 77.27% also attended structured program training.

All those indicating an insufficient level of knowledge and skills participated in structured training. The relationship shown is on the verge of statistical significance (χ^2^ = 5.15; df = 2; *p* = 0.076) (Table 2).

The analysis revealed that among parents who reported having an excellent level of knowledge and skills, 3.85% (2 individuals) participated only in individual sessions, 38.46% (20 individuals) in group sessions, and 57.69% (30 individuals) in both group and individual sessions. In the group of parents with a good level of knowledge, 36.36% (16 individuals) participated in group therapy, while 63.64% (28 individuals) attended both group and individual therapy. Notably, all parents who indicated insufficient knowledge and skills (100%; 4 individuals) participated exclusively in group sessions. This relationship was statistically significant (χ^2^ = 8.18; df = 4; *p* = 0.049) (Table 3).

Based on these findings, it was concluded that participation in individual training is associated with improved knowledge acquisition.

A statistically significant relationship was identified between the frequency of training participation and diabetes-related skills and knowledge (χ^2^ = 32.72; df = 2; *p* < 0.001). All parents with inadequate skill levels participated in training every other day (100%; 4 individuals). In contrast, the majority of parents with excellent skill levels (96.15%; 50 individuals) and those with good competence levels (86.36%; 38 individuals) attended training daily (Table 4).

No significant correlation was found between the level of knowledge and the person conducting the training (χ^2^ = 2.66; df = 4; *p* = 0.614). Across all levels of knowledge, the training was predominantly delivered by a combination of nursing staff, nutritionists, and doctors (Table 5).

The relationship between gender and the level of competence and knowledge about diabetes was examined. The analysis revealed that only women reported insufficient levels of knowledge (100% of those with insufficient knowledge). Among participants with an excellent level of knowledge, 76.92% were women and 23.08% were men. In the group with a good level of knowledge, women comprised 63.64%, while men made up 36.36%. This relationship, however, was not statistically significant (χ^2^ = 3.70; df = 2; *p* = 0.156) (Table 6).

The relationship between age and diabetes-related skills and knowledge was evaluated. No statistically significant association was found between the level of competence and the parents’ age (χ^2^ = 8.51; df = 6; *p* = 0.203). Among parents with an excellent level of knowledge and skills, the majority were aged 25–40 years (57.69%; 30 individuals). In the group indicating a good level of knowledge, 50.00% (22 individuals) were aged 25–40, and 45.45% (20 individuals) were aged 41–55. All parents with an inadequate level of knowledge were within the 25–40 age group (Table 7).

The relationship between the level of education and diabetes-related skills and knowledge was assessed, with the results presented in Table 8. It was observed that individuals with higher education predominantly reported a very good or good level of knowledge. In the group with an excellent level of knowledge, 50.00% had higher education, while among those with a good level of knowledge, 40.91% held higher education degrees. Conversely, parents with only a high school education were more likely to demonstrate insufficient knowledge. However, this relationship was not statistically significant (χ^2^ = 6.92; df = 4; *p* = 0.140) (Table 8).

## 5. Discussion

In the current era, where health behavior knowledge and personal responsibility for one’s health are emphasized, education plays a crucial role in managing chronic diseases like type I diabetes. Parents’ knowledge and skills in managing their children’s type I diabetes significantly influence both the effectiveness of therapy and the quality of life for patients. As a result, the educational process has become an essential component of comprehensive care for children and adolescents with type I diabetes [14,15].

The study conducted in this analysis aimed to evaluate the effectiveness of the educational process and identify factors that influence parents’ knowledge and skill levels. The analysis considered several research aspects, including the form of training, the frequency of participation, the location, and the relationship between knowledge levels and variables such as gender, age, and the education level of the parents. In today’s healthcare landscape, where knowledge of health behaviors and personal responsibility are emphasized, education plays a vital role in managing chronic diseases like type 1 diabetes. As this study shows, parental education is critical in ensuring effective diabetes management for children and adolescents, ultimately improving therapy outcomes and quality of life. This research highlights that factors such as the format and frequency of training, as well as the involvement of a multidisciplinary team, are pivotal in enhancing parents’ knowledge and skills. This aligns with findings from other studies indicating that structured educational programs can lead to better glycemic control, fewer emergency incidents, and improved quality of life. Integrating modern technology into these educational programs, particularly ICT-based therapeutic education, further enhances their impact, with notable reductions in HbA1c levels and the risk of complications [16].

The findings from this study demonstrate that therapeutic education plays a pivotal role in enhancing parental knowledge about type I diabetes. Before their child’s diagnosis, only a small percentage of parents had any understanding of the disease. However, participation in educational programs significantly improved their knowledge, indicating the effectiveness of these interventions. Similar outcomes were reported by Romero-Castillo et al. and Haas et al., whose studies confirm that diabetes patients who engage in structured educational programs show improved blood glucose control, experience fewer hypoglycemic events, make fewer emergency calls and hospital visits, and enjoy better quality of life. However, many still struggle with maintaining self-management [5,17].

Research indicates that ICT-based therapeutic education can lead to a significant reduction in hemoglobin A1c (HbA1c) levels by 0.6%, corresponding to a 17% decrease in the risk of diabetic complications when compared to traditional therapeutic education, as reported by Gomez et al. [18]. In a digital age, where parents of young children with diabetes typically belong to a younger demographic, the integration of modern technologies into education—including therapeutic education—appears essential. This group is generally comfortable with digital solutions, as demonstrated by our study findings regarding the operation of glucometers. This technological proficiency may also enhance cost-effectiveness, as noted by Klonoff and David [19].

Furthermore, the analysis reveals that the form of training, the frequency of attendance, and the location can significantly impact parents’ levels of knowledge and skills. Training provided by a multidisciplinary team of nurses, dietitians, and physicians received positive feedback from the majority of respondents, suggesting that a collaborative approach is crucial for an effective educational process. This finding is consistent with the studies conducted by Fischer et al. and Celik et al. [20,21].

Previous research has shown that nutrition education interventions can lead to significant reductions not only in body mass index (BMI) and HbA1c but also fasting blood sugar levels and the risk of microvascular complications and cardiovascular diseases. Therefore, it can be concluded that the quantity and frequency of training sessions can significantly enhance the effectiveness of the educational process, as highlighted by respondents and supported by studies conducted by Kim and Hur [3].

Additionally, our study revealed significant correlations between education level and diabetes knowledge. Individuals with higher education levels tended to possess better knowledge, likely due to a greater inclination to seek out and assimilate information. However, no significant correlations were found between knowledge levels and gender or age, indicating that these factors do not substantially influence knowledge about type I diabetes. Other studies, including those conducted by Liao et al. and Karakurt et al., also suggest a positive relationship between education level and diabetes prognosis and the effectiveness of treatment program implementations [22,23].

Moreover, there is a noteworthy relationship between the frequency of training participation and levels of skills and knowledge. Individuals who attended training daily tended to demonstrate a higher level of knowledge, emphasizing the importance of regular involvement in the educational process. This finding aligns with the documentation provided by Lima de Melo et al. and Ayar [24,25].

## 6. Conclusions

In conclusion, the findings of this study affirm the effectiveness of the educational-therapeutic process for parents of children and adolescents with type I diabetes. Factors such as the format, frequency, and location of training, along with the involvement of a multidisciplinary team, significantly contribute to the success of the educational process. Future research should aim to identify optimal educational strategies that enhance knowledge and skills for effective diabetes management in young patients.

One key area of focus is enhancing training accessibility. Expanding the availability of training programs to provide a more personalized approach to each child’s education on type I diabetes is essential. This could involve increasing the number of specialists qualified to deliver such training and offering flexible scheduling to accommodate individual meetings. Additionally, increasing the frequency of training sessions is beneficial for reinforcing parents’ knowledge and skills. Regular and frequent educational meetings would allow parents to deepen their understanding of type I diabetes systematically, a goal that could be supported by establishing regular training cycles and ensuring access to online educational resources.

Another focus should be on targeted support for parents with secondary education, as this group often reports a lower baseline level of knowledge. Tailored training programs that meet the specific needs and comprehension levels of these parents are advisable, with educational materials presented in a clear and accessible format to support effective learning.

Continued research in this field is essential for a better understanding of the factors influencing the education of parents of children with type I diabetes. Ongoing studies could help pinpoint areas requiring additional support or adjustments in educational strategies. Implementing strategic changes based on these findings can further improve the educational-therapeutic process for children and adolescents with type I diabetes by enhancing parents’ knowledge and competencies while refining training to meet their specific needs. Concurrently, ongoing investigation into the factors impacting knowledge and skill levels will contribute to the continuous improvement of the educational process.

## Figures and Tables

**Table 1:** Relationship between the place of starting education and the level of knowledge and skills in diabetes management (N = 100).

	Place Where Education Begins	Skill Level			Total
		Excellent	Good	Insufficient	
Number	Outpatient clinic	10	2	0	12
% of the column		19.23%	4.55%	0.00%	
Number	In the diabetology department	40	32	4	76
% of the column		76.92%	72.73%	100.00%	
Number	Other places	2	10	0	12
% of the column		3.85%	22.73%	0.00%	
Total number		52	44	4	100
χ^2^ = 12.73; df = 4; *p* = 0.012					

Source: Authors’ own elaboration based on the survey.

**Table 2:** Relationship between the education model and the level of knowledge and skills in diabetes management (N = 100).

	Education Model	Skill Level			Total
		Excellent	Good	Insufficient	
Number	Transmitted information occurs in an unplanned manner	4	10	0	14
% of the column		7.69%	22.73%	0.00%	
Number	In a structured program training	48	34	4	86
% of the column		92.31%	77.27%	100.00%	
Total number		52	44	4	100
χ^2^ = 5.15; df = 2; *p* = 0.076					

Source: Authors’ own elaboration based on the survey.

**Table 3:** Relationship between the form of training participation and diabetes-related knowledge and skills (N = 100).

	Form of Participation in Training	Skill Level			Total
		Excellent	Good	Insufficient	
Number	Individual	2	0	0	2
% of the column		3.85%	0.00%	0.00%	
Number	Group	20	16	4	40
% of the column		38.46%	36.36%	100.00%	
Number	Individual and group	30	28	0	58
% of the column		57.69%	63.64%	0.00%	
Total number		52	44	4	100
χ^2^ = 8.18; df = 4; *p* = 0.049					

Source: Authors’ own elaboration based on the survey.

**Table 4:** Relationship between frequency of training attendance and diabetes-related skills and knowledge (N = 100).

	Frequency of Attendance	Skill Level			Total
		Excellent	Good	Insufficient	
Number	Once a day	50	38	0	88
% of the column		96.15%	86.36%	0.00%	
Number	Every second day	2	6	4	12
% of the column		3.85%	13.64%	100.00%	
Total number		52	44	4	100
χ^2^ = 32.72; df = 2; *p* < 0.001					

Source: Authors’ own elaboration based on the survey.

**Table 5:** Relationship between training provider and diabetes-related skills and knowledge (N = 100).

	People in Charge	Skill Level			Total
		Excellent	Good	Insufficient	
Number	The nursing team	8	8	0	16
% of the column		15.38%	18.18%	0.00%	
Number	The nutritionists’ team	4	6	0	10
% of the column		7.69%	13.64%	0.00%	
Number	A team of nurses, nutritionists, and doctors	40	30	4	74
% of the column		76.92%	68.18%	100.00%	
Total number		52	44	4	100
χ^2^ = 2.66; df = 4; *p* = 0.614					

Source: Authors’ own elaboration based on the survey.

**Table 6:** Relationship between gender and the level of knowledge and skills in diabetes management (N = 100).

	Gender	Skill Level			Total
		Excellent	Good	Insufficient	
Number	Female	40	28	4	72
% of the column		76.92%	63.64%	100.00%	
Number	Male	12	16	0	28
% of column		23.08%	36.36%	0.00%	
Total number		52	44	4	100
χ^2^ = 3.70; df = 2; *p* = 0.156					

Source: Authors’ own elaboration based on the survey.

**Table 7:** Relationship between age and the level of knowledge and skills in diabetes management (N = 100).

	Age	Skill Level			Total
		Excellent	Good	Insufficient	
Number	Less than 25 years of age	4	0	0	4
% of the column		7.69%	0.00%	0.00%	
Number	25–40 years of age	30	22	4	56
% of the column		57.69%	50.00%	100.00%	
Number	41–55 years of age	16	20	0	36
% of the column		30.77%	45.45%	0.00%	
Number	Over 55 years of age	2	2	0	4
% of the column		3.85%	4.55%	0.00%	
Total number		52	44	4	100
χ^2^ = 8.51; df = 6; *p* = 0.203					

Source: Authors’ own elaboration based on the survey.

**Table 8:** Relationship between education level and the level of knowledge and skills in diabetes management (N = 100).

	Education Level	Skill Level			Total
		Excellent	Good	Insufficient	
Number	Vocational school	8	6	0	14
% of the column		15.38%	13.64%	0.00%	
Number	Secondary education	18	20	4	42
% of the column		34.62%	45.45%	100.00%	
Number	Higher education	26	18	0	44
% of the column		50.00%	40.91%	0.00%	
Total number		52	44	4	100
χ^2^ = 6.92; df = 4; *p* = 0.140					

Source: Authors’ own elaboration based on the survey.

## Data Availability

The datasets generated and analyzed during the current study are available from the corresponding author.
